# Representation Learning Method for Circular Seal Based on Modified MLP-Mixer

**DOI:** 10.3390/e25111521

**Published:** 2023-11-06

**Authors:** Yuan Cao, You Zhou, Zhiwen Zhang, Enyi Yao

**Affiliations:** 1 College of Information Science and Engineering, Hohai University, Changzhou 213022, China; caoyuan0908@gmail.com (Y.C.); zy18013655676@163.com (Y.Z.); zzwloveslife@hhu.edu.cn (Z.Z.); 2 School of Microelectronics, South China University of Technology, Guangzhou 511442, China

**Keywords:** seal recognition, MLP-Mixer, representation learning

## Abstract

This study proposes Stamp-MLP, an enhanced seal impression representation learning technique based on MLP-Mixer. Instead of using the patch linear mapping preprocessing method, this technique uses circular seal remapping, which reserves the seals’ underlying pixel-level information. In the proposed Stamp-MLP, the average pooling is replaced by a global pooling of attention to extract the information more comprehensively. There were three classification tasks in our proposed method: categorizing the seal surface, identifying the product type, and distinguishing individual seals. The three tasks shared an identical dataset comprising 81 seals, encompassing 16 distinct seal surfaces, with each surface featuring six diverse product types. The experiment results showed that, in comparison to MLP-Mixer, VGG16, and ResNet50, the proposed Stamp-MLP achieved the highest classification accuracy (89.61%) in seal surface classification tasks with fewer training samples. Meanwhile, Stamp-MLP outperformed the others with accuracy rates of 90.68% and 91.96% in the product type and seal impression classification tasks, respectively. Moreover, Stamp-MLP had the fewest model parameters (2.67 M).

## 1. Introduction

A seal is a token stamped on a document to indicate a signature or authentication, with the name of the unit or individual engraved on it. The seal is an important tool to confirm the identity of a legal person and plays an important role in daily life. The seal impression is the surface content formed by the seal stamped on paper and other document carriers. The traditional method of identifying forged seals usually identifies the seal by manually comparing the seal impression. This method is not only time-consuming, but also unreliable. Many researchers have applied support vector machines and deep learning methods for the automated verification of seal imprints; however, in actual application scenarios, it is difficult to obtain enough negative samples. Thus, it is difficult for researchers to satisfactorily train machine learning models. Additionally, the limited proportion of negative samples leads to an imbalanced distribution between positive and negative samples, which can result in machine learning classifiers learning a biased decision boundary, effectively classifying all samples as positive [1].

Information technology can help quickly identify seals. How to effectively extract the features of seal impressions has become the focus of research. In the task of seal identification, early work was often based on manually extracted features. References [2,3] used the simple geometric features of the seal to realize the registration of the seal. Reference [4] used the SIFT of the seal impression images to realize the verification of the seal. Reference [5] used a simpler point-matching algorithm to realize the verification of the seal. M. Yao [6] realized the detection, positioning, and registration of seal impressions based on the SIFT features and the RANSAC algorithm. J.S. Liang [7] used the difference image method to perform an XOR operation on the registered seal and the questioned seal and calculated the matching similarity, but the accuracy of this method for identifying fake seals was low. Q. Guo et al. used column sparsity optimization to complete the registration of seals [8]. Y.C. Su et al. used the edge difference to realize the automatic recognition of the seal [9]. T.T. Shao calculated the likelihood ratio through the characteristics of the seal diameter, side width, five-star angle, and five-star distance, and applied it to seal identification [10]. F.W. Liu [11] and Y.H. Xu [12] used a probability-distribution-based seal authenticity recognition algorithm, but this method was not effective for the commonly used circular seal recognition. None of these works could accurately identify fake seals because they only exploited simple manual features. Hand-designed features often have relatively large limitations and are only applicable to specific seal types, and the accuracy rate is also low.

Utilizing Convolutional Neural Networks (CNNs) [13,14,15] to automatically extract impression features shows greater advantages and adaptability. In many works [16,17,18,19], it has become a trend to use CNNs to automatically extract features and generate representations. Reference [20] utilized deep convolutional networks to learn representations of image features for image retrieval. In signature offline verification, many works use CNNs to learn representations of images [21,22,23,24]. In the seal recognition and verification tasks, the method of using CNNs to automatically extract seal features [15] has a higher accuracy. Q. Zhang [25] studied the influence of stamping conditions on the use of CNNs to identify seals and discussed their feasibility and practicability. However, the CNNs used in these works required a large number of samples of the same category for training the network, which lacks practicability.

Recently, Transformer [26,27] has received extensive attention in computer vision. It is completely based on self-attention, abandoning the local information aggregation of the convolutional structure, and has achieved comparable results to CNNs. However, due to a lack of inductive bias, it relies on extensive datasets for training and often struggles with generalization when the data are limited [28]. MLP-Mixer [29,30] based only on Multi-Layer Perceptron (MLP) and residual connections has also achieved surprising results. Due to its simple structure and small model size, more application scenarios can be obtained. MLP-Mixer has a good ability to capture global information and can have an advantage in the identity detection of seal impressions, because the difference between fake seals and real seals is very small, and the model needs to have a stronger ability to extract the underlying features of impressions. The underlying features are widely and evenly distributed in the global image of the seal impressions.

This paper proposes Stamp-MLP, a new representation learning method named for circular seals, which is based on the improved MLP-Mixer. The proposed method uses circular stamp remapping instead of patch segmentation projection. Besides, it introduces a global pooling method based on self-attention to improve the accuracy of model learning representation. Stamp-MLP was compared with MLP, VGG16, and ResNet50 to verify the accuracy and computational complexity with a dataset of 8616 seal impressions, which consisted of 81 different seals. The dataset comprised 16 seal surfaces, each of which had six distinct product types. Compared to MLP-Mixer, VGG16, and ResNet50, across seal surfaces, product types, individual seals, and computational complexity, Stamp-MLP boasted the dual benefits of superior precision and reduced complexity.

## 2. Related Work

Transformer [27] in natural language processing has been successfully introduced into the field of vision. DETR [31] omits the post-NMS processing step of non-maximum suppression, gets rid of the prior knowledge and constraints of anchors, and greatly simplifies the principle of target detection. On this basis, CLS-DETR [32], DN-DETR [33], WB-DETR [34], and other improved DETRs further promote the application of Transformer in the field of vision. ViT [26] replaces the convolution operation in CNNs with multi-head self-attention, which divides the image into fixed-size patches, and uses Transformer’s powerful global information capture capability to model the connections between these patches. MLP-Mixer [29] uses the same preprocessing operation, but it replaces the self-attention in Transformer with a simpler multi-layer perceptron and has also achieved good results. Due to its simple and effective structure, MLP-Mixer greatly simplifies the computational complexity of the model.

However, similar to ViT, MLP-Mixer weakens the aggregation ability of local information. AS-MLP [35], CycleMLP [36], $S^{2}$-MLP [37], and $S^{2}$-MLPv2 [38] all choose to move the axis along the channel to achieve the aggregation of local information and obtain a receptive field similar to CNNs. However, since only the channel mixing MLP is retained, they also lose the global information capture ability of the MLP-Mixer model. AS-MLP and CycleMLP can also be used as backbone networks to replace CNNs to execute target detection and segmentation tasks. ViP [39] also encodes information along the spatial structure, but instead of moving the spatial structure like AS-MLP, it uses a linear projection, which requires more computation in comparison. Different from the above methods, RaftMLP [40] sequentially encodes information serially along the vertical and horizontal directions. Fer-MLP [41] designs a tokenized MLP block that allows it to extract more-convincing features. Hire-MLP [42] proposes inner-region rearrangement and cross-region rearrangement, enabling information communication between different regions and capturing the global context.  Studies [43,44,45] on the connection and robustness of MLP-Mixer, Transformer, and CNNs have shown that MLP-Mixer and Transformer have better adversarial robustness than CNNs. MLP-Mixer has also been explored with some applications. PointMixer [46] is used for point cloud understanding. MS-MLP [47] is used for ECG classification. In [48], MLP-Mixer was used for artistic style classification. Moreover, Wang [49] successfully detected Alzheimer’s disease through MLP. Inspired by Fractional Fourier Entropy’s application to pathological brain detection [50], Zhang [51] developed an MLP-based pathological-brain-detection system by combining MLP and Fractional Fourier Entropy.

In this paper, the remapping operation of the circular seal is input into the MLP, which preserves the underlying pixel information and spatial structure of the seal to the greatest extent and introduces attention-based global pooling to improve the accuracy of the model.

## 3. Proposed Methodology

The proposed approach can be generally divided into two stages: (i) firstly, the circular seal impression is remapped and aligned in a rectangle grayscale image; (ii) secondly, MLP-Mixer is used to classify the seal impression, skipping the step in the traditional MLP-Mixer of splitting the image into patches. Instead, the network receives the remapped seal impression picture directly. Each of the circular seals’ radii is treated as a token and re-stacked into a matrix. These two stages are discussed in detail in the following subsections.

### 3.1. Remap and Alignment

#### 3.1.1. Color Segmentation

**Figure 1:**
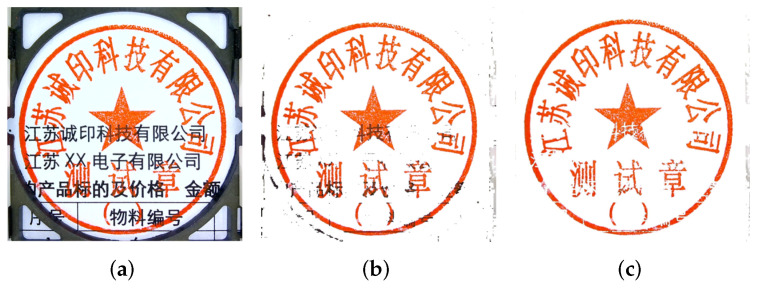
(**a**) The original image. (**b**) Overlapped image after applying Equation (1). (**c**) The final image after color segmentation.

The color segmentation plays a vital role in extracting the region of interest (i.e., seal text) from the background image. Generally, the seal impression has a blank graphic background with the seal text in red, as shown in Figure 1a. The seal impressions are extracted to conform with the RGB color scheme if the RGB pixel value satisfies Equation (1). It should be noted that, in Asian countries, almost all seals are red circular seals, so Equation (1) is very suitable to the experiments in this paper. However, the proposed method is also applicable to other seals with different colors. For instance, if the color of the seal is blue, Equation (1) can be rewritten as Equation (2).

(1) $$\left(R>B\right)\&\left(R>G\right)\&\left(R>150\right)$$

(2) $$\left(B>R\right)\&\left(B>G\right)\&\left(B>150\right)$$

where, *R*, *G*, and *B* define the intensity of the color. The proposed color segmentation initially sets all pixels to white that do not satisfy Equation (1), therefore getting rid of most of the black area in the seal impression (Figure 1b). The rest of the seal impression contains an overlap region of the seal and the black background. The overlapping area between the seal and the background is then filtered out to obtain a clean circular seal impression (Figure 1c). It is worth mentioning that this filtering is performed while retaining the maximum details of the seal impression, because we used the color segmentation algorithm [52] through RGB color space, which is the most-effective color segmentation algorithm and can preserve the feature of the color very well after segmentation.

#### 3.1.2. Hough Circle Detection

Following the color segmentation, the detection of the seal’s center and radius is performed using the Hough Circle Transform. The seal impressions have missing edges. Therefore, locating the perfect circle area becomes a challenging task. This issue is addressed by performing binarization of the color segmented image, which fills the disjoint circular edges and produces a complete circular area. In this work, a 5 × 5 kernel was used, and there were three total expansion operations, which were proven to have the best effect in the experiment.

**Figure 2:**
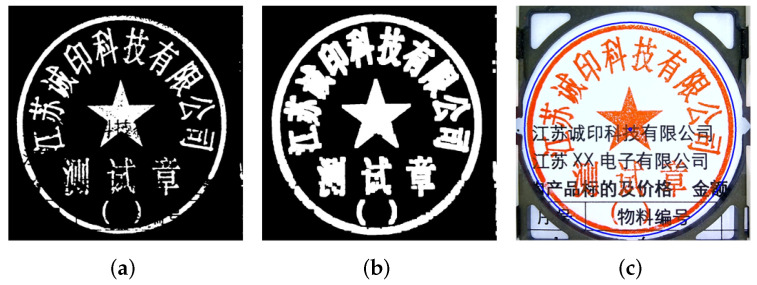
(**a**) The binarized image. (**b**) The image after dilation. (**c**) The result of circle location.

The implementation of the Hough gradient traverses the center of the circle [53], which corresponds to all non-zero pixels, and uses that area for detection. The intersection of all the modulo vectors on the circle is the circle’s center. Each point on the circle has a modulo vector, which is the vertical line that is tangent to that point. The number of modulo vector intersections serves as the basis for the Hough gradient method’s judgment. Figure 2 illustrates this with the binarized image on the left, the dilation-affected binarized image in the middle, and the circle detection result on the right.

#### 3.1.3. Remap to Matrix

The Hough Transform provided us with the location of the circular seal, and as a result, we obtained an accurate seal image without any black background. The next step was to effectively map the seal impression to a matrix.

**Figure 3:**
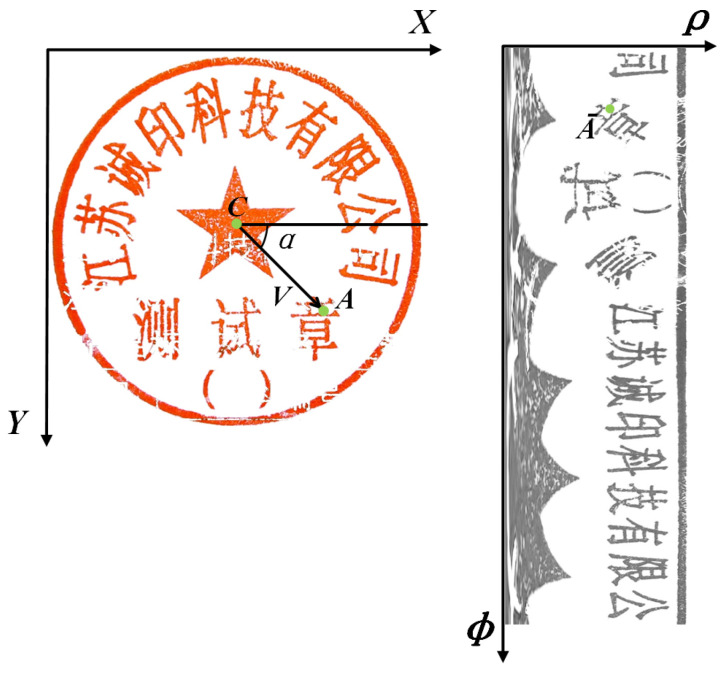
Demonstrates the process of mapping pixels from a circular area to a matrix.

Figure 3 illustrates the mapping of the circular seal picture to a single-channel rectangle image. To create a new matrix, the original MLP-Mixer model separates the left image input into S × S grids, linearly projects each grid into a vector, and then, stacks the S${}^{2}$ grid representations. We enhanced it by remapping the circular seal to a matrix because the original action will cause the representation of the printed text to lose many features.

Initially, the original image was remapped from the polar coordinate to the Cartesian coordinate using Equation (3) [54], where *C* is the center of the seal, *A* is a point in the left image, and $\bar{A}$ is the corresponding point after *A* is remapped. This remapping ensures that the area remains the same; therefore, the width of the rectangle is the same as the radius of the circle, and the height of the rectangle is π times the radius.

(3) $$\begin{array}{c} \;\;\;\;\;\;\;\;\;\;\;\;\;C\left(x_{c},y_{c}\right),A\left(x,y\right),\bar{A}\left(\rho ,\phi \right)\\ V=\left(x-x_{c},y-y_{c}\right)\\ \;l=magnitude\left(V\right),a=angle\left(V\right)\\ \;\;\;width=R,height=R\cdot \pi \\ \;\;\;\;\;\;\rho =\frac{l}{R}width,\phi =\frac{a}{2\pi }height \end{array}$$

#### 3.1.4. Alignment

**Figure 4:**
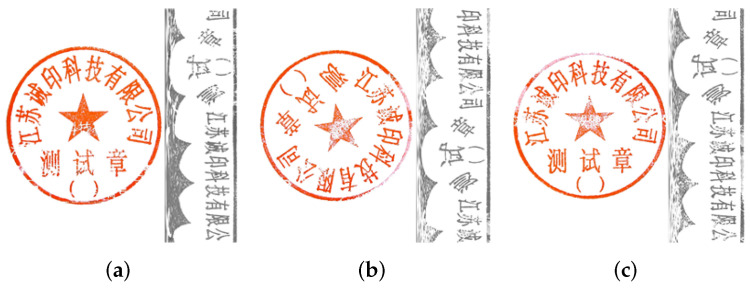
(**a**) The standard seal. (**b**) The seal to be aligned. (**c**) The aligned seal image.

In this step, the elements of the seal impression are extracted by using the seal pixel information. This task becomes challenging because the orientation of the circular seal varies with the method by which the seal picture is acquired. Therefore, we propose an alignment technique where the remapped rectangular grayscale image (obtained from the above section) is aligned with a standard seal image, as shown in Figure 4. The offset calculation and the rotation operation were used to perform this alignment effectively.

**Figure 5:**
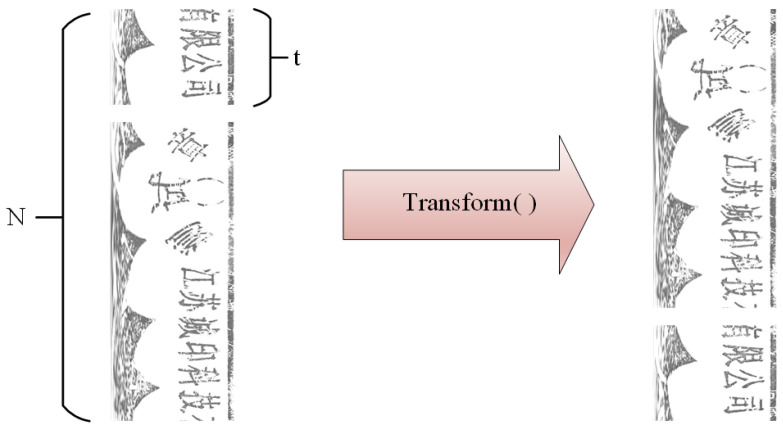
The rolling process of the matrix is shown, which corresponds to the rotation of the circular seal, through which the offset of the two seals can be calculated.

Initially, the seal image is rotated by cutting and rearranging the row vectors of the matrix, as shown in Figure 5. The offset error *E* is defined as Equation (4), where *X* is the image to be aligned and *C* is the corresponding standard image. It can be observed from the formula that, when the offset error *E* is smaller, the image *X* is more like the standard image *C*, and the directions of *X* and *C* tend to be the same.

(4) $$E=Sum\left(\left|X-C\right|\right)$$

Transform (X, t) is the roll transformation function, as shown in Figure 5, where t is the offset, and the first t rows of X are cut and stacked at the end of the row.

The offset algorithm is shown below (Algorithm 1). The image that needs to be aligned is cycled through transformations, and the offset error is determined to obtain the offset error that is the least. The offset between the image that needs to be aligned and the reference image is known as the matching offset. The number of rows in X is equal to the number of cycles N. Finally, we may rotate the circular seal and determine the offset angle. The number of loops and the computational cost can be decreased if the error is permitted by increasing the step size of each transformation above 1.

The purpose of the alignment step was to improve the accuracy of the detection results by aligning the seal images in the same direction. Due to the concern that the use of augmentation will lead to the distortion of the seal image, which will degrade the accuracy of the defect detection, we did not use augmentation technology.
**Algorithm 1** The offset algorithm.**Input: X: image to be aligned; C: the standard image** **Output: offset; OffsetAngle; W**   1:$E_{min}⇐Sum(|X-C|)$;  2:offset⇐0;  3:N⇐Height(X);  4:**for** t=1 to *N* **do**  5:    Y⇐Transform(X,t);  6:    E⇐Sum(|X−C|);  7:    **if** $E<E_{min}$ **then**  8:        $E_{min}⇐E$;  9:        offset⇐t;  10:   **end if**  11:**end for**  12:OffsetAngle⇐offset∗2π/N;  13:W⇐Transform(X,offset);

Circle seal rotation at any angle is achieved by an affine transformation shown in Equation (5) [55]. Here, the angle is the offset angle; ($x_{c}$, $y_{c}$) is the rotation center point; M is the rotation matrix; T() is the rotation transformation function, and the coordinates of each pixel in the original image are transformed to obtain the coordinates in the new image.

(5) $$\begin{array}{c} M=\left[\begin{array}{ccc} \alpha & \beta & \left(1-\alpha \right)\cdot x_{c}-\beta \cdot y_{c}\\ -\beta & \alpha & \beta \cdot x_{c}+\left(1-\alpha \right)\cdot y_{c} \end{array}\right]\\ \;\;\alpha =\cos\left(angle\right)\\ \;\;\beta =\sin\left(angle\right)\\ \;\;T\left(\left[\begin{array}{c} x\\ y \end{array}\right]\right)=\left[\begin{array}{c} m_{11}\cdot x+m_{12}\cdot y+m_{13}\\ m_{21}\cdot x+m_{22}\cdot y+m_{23} \end{array}\right] \end{array}$$

### 3.2. The Proposed Architecture

#### 3.2.1. Mixer Layer

**Figure 6:**
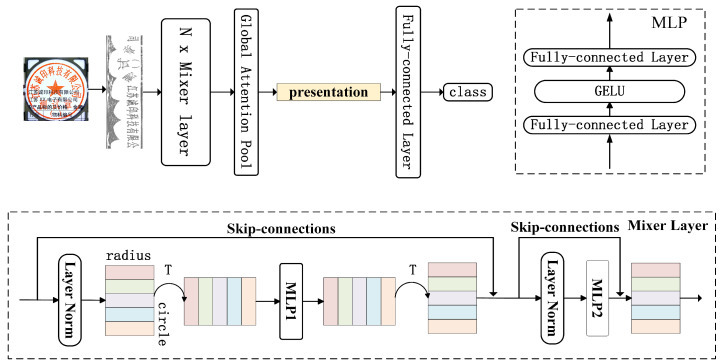
The structure of MLP-Mixer. MLP1 refers to the MLP in the circumferential direction; MLP2 refers to the MLP in the radial direction.

**Figure 7:**
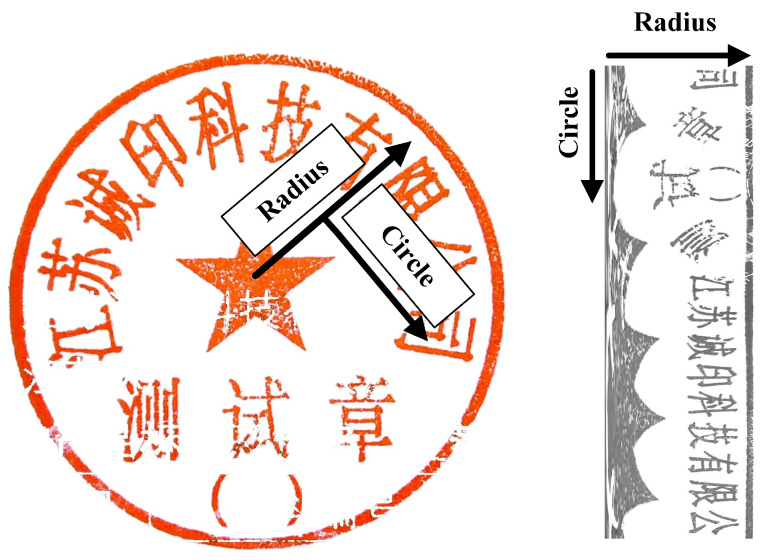
Mixer layer aggregates the seal features along with the radius and circle.

Figure 6 depicts the structure of MLP-Mixer, which is made by stacking many Mixer layers. The MLP in the radial direction and the MLP in the circumferential direction are both present in the Mixer layer, which is applied to the remapped single-channel image. Two fully connected layers and nonlinear activation functions make up each MLP construction block. The two MLPs operate independently of one another in different directions. The first MLP works in the radial direction to aggregate features by taking into account the input X’s columns, while the second MLP works in the circumferential direction by taking X’s rows as the input to accumulate the features (Figure 7). Additionally, skip connections are established for every MLP. Before the input to MLP, normalization is performed using LayerNorm.

The mathematical representation of the Mixer layer is shown in Equation (6), where X is the remapped seal; U and Y are MLP1’s output and MLP2’s output, respectively. X, U, and Y have the same shape due to the use of skip connections. MLP1 and MLP2 are the circumferential and the radial direction MLPs, respectively. Here, $W_{i}$ and $b_{i}$ are the weights and biases of the fully connected layer, and σ refers to the GELU nonlinear activation function. It needs to be stressed that image augmentation technology is not applied in this model, because if we use the image augmentation technology like slice, many seal surface images will not match the reality. For example, there is no such thing as a half seal surface in real life. Moreover, if we apply image augmentation technology like mosaic [56] and mixup [57], these methods can seriously damage the structure of seal surfaces, which is not conductive to the identification of forged seals.

(6) $$\begin{array}{cc} \; & U=X+{MLP}_{1}{\left(LayerNorm{\left(X\right)}^{T}\right)}^{T}\\ \; & Y=U+MLP_{2}\left(LayerNorm\left(U\right)\right)\\ \; & MLP_{1}\left(X^{T}\right)=\sigma \left(X^{T}W_{1}+b_{1}\right)W_{2}+b_{2}\\ \; & MLP_{2}\left(U\right)=\sigma \left(UW_{3}+b_{3}\right)W_{4}+b_{4} \end{array}$$

#### 3.2.2. Attention-Based Global Pooling

In this paper, a global pooling method based on attention was designed to replace the global average pooling in the original MLP-Mixer. The specific method is shown as Equation (7), where X is the feature map obtained from the Mixer layer feature aggregation. After pooling, we aimed to achieve a vector that is the representation of the seal impression. The dimension of the representation is W, and the global average pooling averages X along the column. Our method generates a set of weights for each row of X, and the weighted average is obtained along the column. Here, $W_{Q}$ is the learnable parameter, Q is the query, and the product S of the transposition of Q and X is the score, which is converted into the weight value of each row vector of the input X through the softmax function. The final output value V is the representation of the globally pooled seal representation.

(7) $$\begin{array}{c} Q=W_{Q}\times X,X\in R^{h\times w},Q\in R^{1\times w}\\ \;\;S=Q\times X^{T},S\in R^{1\times h}\\ \;\;\;\;V=soft\max\left(S\right)\times X,V\in R^{1\times w} \end{array}$$

## 4. Results and Experiments

### 4.1. Dataset and Experiment Setup

**Figure 8:**
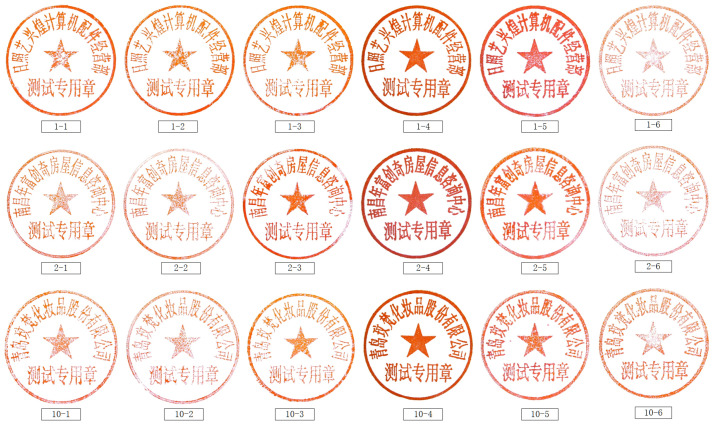
The seal impression images of 3 seal surfaces (1, 2, 10) for 6 product types. Seal surface means a seal with different characters, and product type refers to the different processes of production.

**Figure 9:**
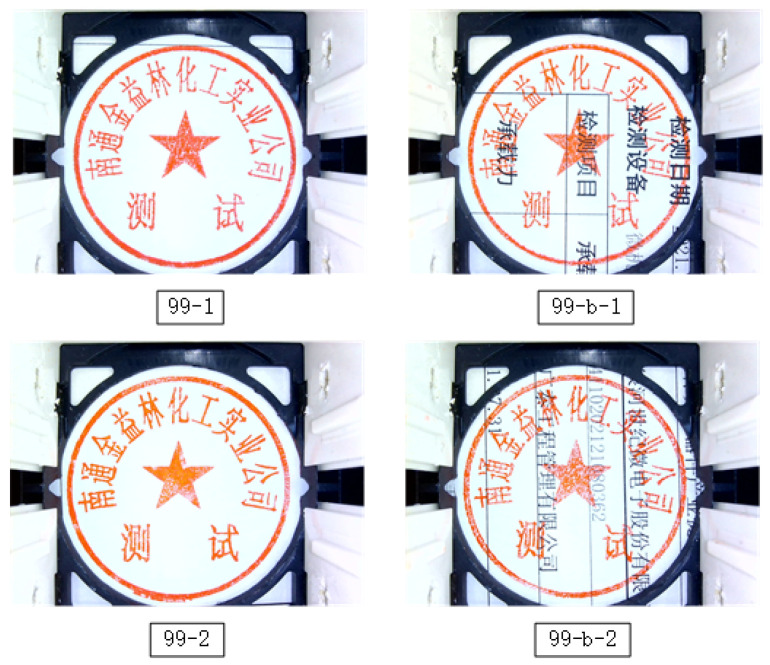
Here, “99-b” refers to the image with a black text background, which adds noise to the seal’s features.

Dataset: For a fair comparison, an indigenously designed dataset of 81 seals was used in the experiments. This dataset contained 16 seal surfaces, and each seal surface had six different product types. All 16 seal surfaces had different character formations, and the six product types of each seal surface corresponded to the fact that each one was produced by different manufacturers (Figure 8). It is pertinent to mention that all the following factors led to producing six different product types of each seal surface: (i) the seal templates, (ii) the materials and machinery used to produce the seal, (iii) the manufacturing processes. Moreover, the manufacturer, materials, and equipment used to create product types −1, −2, and −3 were from the same company. The seals created with Types −1, −2, and −3 look quite like one another; however, they are different. Contrarily, the types −4, −5, and −6 were assumed to be forged seals. They were produced by different manufacturers by employing various production techniques and materials. Figure 8 shows the photos of three seal surfaces (numbered 1, 2, and 10) with six different product types. Table 1 displays the total number of pictures gathered for each seal, which comes to 8616. One of these, 99-b, refers to the background image that was gathered, as seen in Figure 9. In the experiments, we used background-free photos for training and background-containing images as a test set to assess how robust the approach was to background noise.

The efficacy of our proposed model was examined from various perspectives by dividing the experiment into the following three classification tasks: (i) classifying the seal surface, (ii) the product type, and (iii) the individual seal. The three classification tasks used an identical dataset with different targets of 16 classes, 6 classes, and 81 classes. The first 48 photographs of each seal were used as the training sets, while the remaining images were used as the test sets. According to Table 2, there were 3888 total training sets. For the classification job of the seal surface, we only used the 72 photos of Product Type −5 for training and compared the classification results to further confirm the method’s generalizability.

Metrics: The top-1 and top-3 accuracy metrics were used to estimate the performance of the proposed model. To balance the number of samples, the sample weight parameter was added to the calculation, then the computational complexity and parameter count of the model were compared concurrently.

Model: The 3- and 6-layer Mixer layer models were designed and named StampMLP-3 and StampMLP-6, respectively. The models with a different number of layers helped to explore the impact of the number of layers on the accuracy when compared with MLP-Mixer-3/6, VGG16, and ResNet50. MLP-Mixer and Stamp-MLP have the same hidden layer dimensions (the channel-dim was 128, and the token-dim was 512). However, the input resolutions were different: in Stamp-MLP, the resolution was 798 × 256, while it was 512 × 512 for MLP-Mixer (with a grid resolution of 16 × 16), VGG16, and ResNet50.

The Pytorch library was used to design Stamp-MLP, and the experiments were performed using the Nvidia RTX3090 and the Adam optimizer. It is pertinent to mention that all the experiments were performed with the following default settings: a learning rate of 0.001, a batch size of 128, and a number of training epochs of 48.

### 4.2. Result

In order to prevent the influence of overfitting, the data in all the results were obtained when the accuracy of the test set and the training set were the closest.

#### 4.2.1. Product Type Classification

The classification accuracy is displayed in Table 3, where Top-1* denotes the first three product types (i.e., −1, −2, and −3) belonging to the same category because their manufacturing procedures were identical. All the preprocessing operations were applied for all the models in our experiments, except the remap operation, which is one of our novelties and was only applied on our model. It is evident from Table 3 that Stamp-MLP’s top-1 accuracy was better than VGG16’s, MLP-Mixer’s, and ResNet50’s. Moreover, the proposed strategy slightly was more resistant to the test set with background noise.

Since the disparities between the first three classes (i.e., −1, −2, and −3) were the smallest, the misclassification was mostly centered on these three. However, as Stamp-MLP has a stronger ability to capture pixel-level features, it classified these three classes better than the existing approaches.

#### 4.2.2. Seal Surface Classification

The character information in the seals was used by all the models in surface classification. As shown in Table 4, VGG16, ResNet50, and Stamp-MLP attained accuracy levels greater than 99%, while MLP-Mixer had an accuracy under 99%. However, on the test set with the background noise, all models had a 100% classification accuracy.

The quantity of the training sets was significantly reduced to evaluate the models under difficult conditions. To evaluate the model’s capacity in handling smaller datasets, we used 72 types −5 print images as the training sets. The experimental findings are shown in Table 5. While all four models’ accuracy decreased to some degree, VGG16 and ResNet50 were highly affected. The top-1 accuracy decreased by more than 19%, while for Stamp-MLP, the accuracy decreased by only 10%.

#### 4.2.3. Individual Seal Classification

The model must be able to capture the distinctive characteristics that each seal-making technique produces to properly identify each seal. Therefore, assessing how well the model performed in terms of classifying individual seals is of utmost importance. Table 6 displays the testing results. Especially when compared to MLP-Mixer, Stamp-MLP had better classification accuracy with 91.96% top-1, 97.87% top-3, and 98.5% BG-top-1. The lowest, MLP-Mixer-6, in top-1* also attained an accuracy of 96.93%, which was greater than the top-1 accuracy of 84.25%.

The first three product kinds were very identical and had very small distinguishing traits; therefore, to properly classify, the underlying pixel-level information was needed. The patch linear projection used in MLP-Mixer resulted in the loss of the underlying data. However, Stamp-MLP retained the maximum pixel-level data, therefore giving in an advantage in identifying the variances existing in the various product types. Moreover, Stamp-MLP was more reliable when dealing with the background.

We also compared the number of parameters, FLOPs, throughput (based on RTX3090), MACs (the batch size was 1), and training epochs in addition to the classification accuracy. Table 7 demonstrates that Stamp-MLP used fewer parameters, required fewer training cycles, used less memory, and had fewer FLOPs. Moreover, the detailed specifications of the implemented environment are shown in Table 8, where “True” indicates we used a GPU.

VGG16’s classification performance in the aforementioned tasks was quite similar to Stamp-MLP; however, Stamp-MLP had the advantage of being more lightweight because it used fewer parameters and fewer optimization epochs, making it better suited for small datasets. Moreover, the performances of the 3-layer model and the 6-layer model were identical, and in practical applications, the 3-layer model can be used to keep the model’s complexity low.

### 4.3. Discussion

Generally, MLP-Mixer divides the projection grid such that much underlying pixel-level information is lost. It was evident from the experimental results that MLP-Mixer’s product type classification was not encouraging; however, the proposed modified MLP-Mixer remaps the pixel positions such that the circular seal is directly passed to the neural network, preserving the maximum usable information. The model extracts the features of the seal imprint by using the feature aggregation of the two MLPs and global pooling and, then, creates a representation of the seal picture.

Both MLP-Mixer and Stamp-MLP can perform better when classifying seal surfaces. Since the counterfeiting of seals is a significant problem in society, it is crucial to preserve and extract the fundamental characteristics of the seal. These characteristics serve as the foundation for further development of forged seal recognition. Forged seals often share high-level characteristics with genuine seals, such as the same characters, size, layout, etc. The underlying pixels of the image contain the data that may be used to identify a false seal. Therefore, the ability to store and recover such pixel-level information is very crucial.

The experimental results showed that the individual seal categorization was most challenging for the differentiation between the first three product type classes, because all three of these product kinds were produced by the same methods. Although these seals were quite similar to each other, in practical scenarios, they are quite difficult to make by the seal’s creator.

The experimental result showed that the CNNs represented by VGG16 behave similarly in classification; however, they were less resistant to noise in the background. CNNs also require complicated feature computations and large parameters. Moreover, CNNs are difficult to optimize.

**Figure 10:**
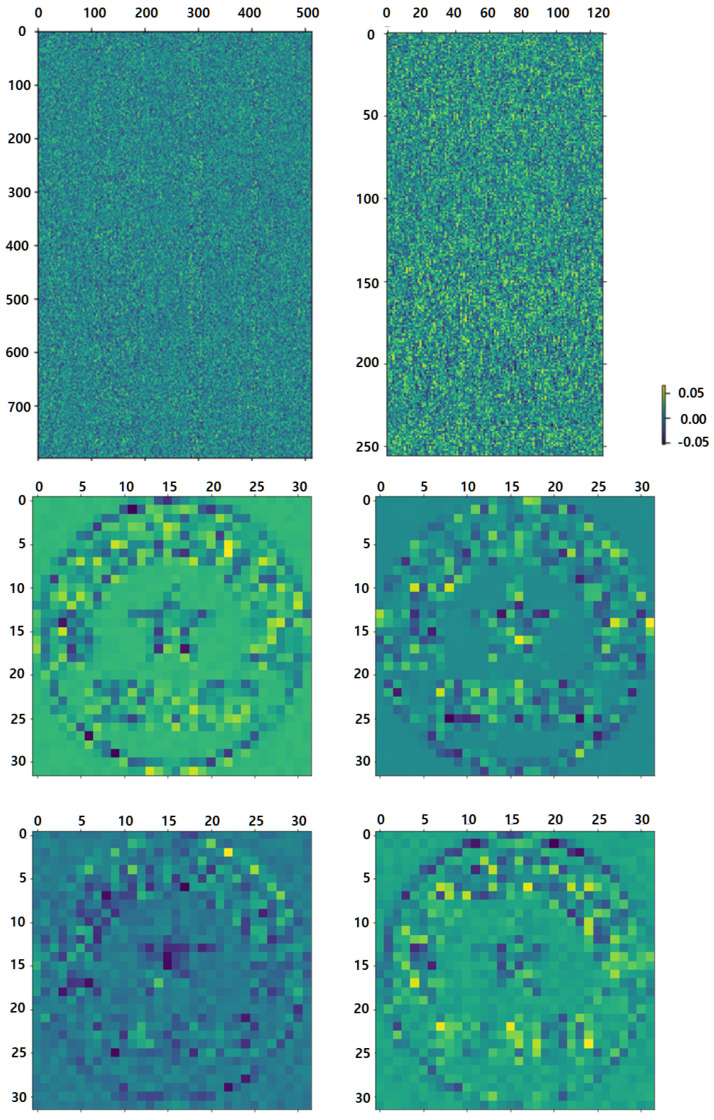
Visualization of the first layer weights of MLP. The upper left is the weight of the first layer of MLP1, and the upper right belongs to MLP2 of our method. The bottom is the first layer of MLP1 of MLP-Mixer with 4 of 512 channels.

The weights of an MLP’s first layer are shown in Figure 10. Stamp-MLP’s MLP1 weight of the first layer is shown in the upper left, and the weight in the upper right belongs to MLP2. The first layer of MLP1 in MLP-Mixer had a total of 512 channels, and the weights for 4 of these channels are shown in the lower layer. Stamp-MLP considers the pixel-level details in the overall image as seen by the uniform and erratic weight distribution. The weights of some channels are significantly larger, and the figure shows some light and dark streaks if the model pays more attention to the information in particular locations. The accuracy of Stamp-MLP was higher because the input data fully preserved the pixel-level information and the model uniformly considers all of the input data.

MLP-Mixer also uses the information included in the seal imprint area, the lettering, and the five-pointed star, as shown in Figure 10. However, the model loses much pixel-level information in the grid (due to the usage of grid split and linear projection), impacting the extraction of grid border information. Finally, this resulted in lower classification accuracy.

What is more, we used two other popular methods for identifying forged seals—directly applying the CNN to the input seal images without our mapping step and using the CNN with augmentation instead of alignment, to perform a simple experimental comparison with our method. We used VGG16 and rotated our seal images at random angles by using data augmentation.

In the experiment to classify product types (six types), we put 3888 seal images directly into VGG16 without our mapping step and achieved 89.84% accuracy after training. Then, we applied data augmentation to increase the number of seal images from 3888 to 10,000 and achieved 92.71% accuracy after training. The classification accuracy is displayed in Table 9, where VGG16 refers to directly applying the CNN to the input seal images without our mapping step. VGG16-1 refers to using the CNN with augmentation instead of alignment. VGG16-2 refers to using the method we proposed in the Proposed Methodology Section. Stamp-MLP-3 and Stamp-MLP-6 refer to our proposed models using the data preprocessing method in the Proposed Methodology Section.

In classifying individual seals (81 classes), we put 3888 seal images directly into VGG16, and after training, we had an accuracy of 88.67%. Then, we applied data augmentation to increase the number of seal images from 3888 to 1000, and the accuracy of VGG16 was 91.21%. The concrete data are shown in Table 10, where VGG16 refers to directly applying the CNN to the input seal images without our mapping step. VGG16-1 refers to using the CNN with augmentation instead of alignment. VGG16-2 refers to using the method we proposed in the Proposed Methodology Section. Stamp-MLP-3 and Stamp-MLP-6 refer to our proposed models using the data preprocessing method in the Proposed Methodology Section.

In the seal surface classification task (16 classes), in order to avoid the accuracy reduction by the difference of the product type, we only chose Product Type −5 for training. Firstly, we put our 1152 seal images directly into VGG16 without the mapping step, and the accuracy was 94.84%. Then, we used data augmentation to increase the number of seal images in the training set from 1152 to 4500 and put them into VGG16. The accuracy was 95.21%. The concrete data are shown in Table 11, where VGG16 refers to directly applying the CNN to the input seal images without our mapping step. VGG16-1 refers to using the CNN with augmentation instead of alignment. VGG16-2 refers to using the method we proposed in the Proposed Methodology Section. Stamp-MLP-3 and Stamp-MLP-6 refer to our proposed models using the data preprocessing method in the Proposed Methodology Section.

### 4.4. Limitations

It is essential to acknowledge that this study has limitations. It solely examined red circular seals, while real-world situations involve seals of different colors and shapes. The limited data availability prevented us from including other types of forged seals, a common challenge in defect detection research. Additionally, external factors like light were not considered in the study, which may have caused interference.

## 5. Conclusions

In this study, a dataset comprising 81 seals was created, encompassing 16 distinct seal surfaces, with each surface featuring six diverse product types. This dataset can become a valuable resource for visual detection studies concerning the discernment of counterfeit seals. A novel representation learning method based on a modified MLP-Mixer was proposed for the identification of circular seals. To maintain the additional pixel-level information, which is crucial for the identification of seals, we remapped the circular seals and replaced the grid split in MLP-Mixer. Moreover, the use of an attention-based global pooling approach made our proposed method lightweight and more accurate. During the experimentation phase, Stamp-MLP exhibited strong performance across all three tasks, showcasing the highest accuracy when applied to scenarios involving test sets with backgrounds, a setting more similar to real-world situations. This highlights Stamp-MLP’s aptitude for effectively detecting forged seals within our everyday contexts. In the future, this study can be extended to investigate identifying imitation seals.

## Figures and Tables

**Table 1:** The number of images collected for each seal.

Seal Surface	Seal Type					
	−1	−2	−3	−4	−5	−6
1	120	120	120	72	72	72
2	120	120	120	72	72	-
3	120	120	120	-	72	72
4	120	120	120	-	72	72
5	120	120	120	-	72	72
6	72	72	72	-	72	72
7	72	72	72	-	72	72
8	72	72	72	-	72	72
9	72	72	72	-	72	72
10	72	72	72	72	72	72
11	72	72	72	-	72	72
12	72	72	72	-	72	72
13	72	72	72	-	72	72
14	72	72	72	-	72	72
15	72	72	72	-	72	72
99	1004	1004	-	-	72	72
99-b	100	100	-	-	-	-

**Table 2:** The number of training sets and test sets. “C16-72” means the classification of the seal surface with only 72 images of the seal for training.

	Training Sets	Test Sets
C16-72	1152	7464
Others	3888	4728

**Table 3:** Product type classification accuracy. Top-1* refers to considering the first three product types (i.e., −1, −2, −3) as the same category. BG-Top-1 means the top-1 accuracy of the test set with the background. MLP-Mixer-3 and Ours-3 denote the models with 3 Mixer layers.

	Top-1*	Top-1	Top-3	BG-Top-1
VGG16	99.56%	88.99%	99.41%	75.50%
ResNet50	99.00%	69.96%	99.31%	82.50%
MLP-Mixer-3	98.39%	83.93%	98.80%	73.00%
MLP-Mixer-6	98.32%	80.38%	97.82%	66.50%
Stamp-MLP-3	98.96%	90.60%	98.86%	94.00%
Stamp-MLP-6	99.03%	90.68%	98.31%	95.00%

**Table 4:** Accuracy of seal surface classification.

	Top-1	Top-3	BG-Top-1
VGG16	99.97%	100.00%	100.00%
ResNet50	99.60%	99.89%	100.00%
MLP-Mixer-3	98.87%	99.70%	100.00%
MLP-Mixer-6	98.36%	99.45%	100.00%
StampMLP-3	99.47%	99.74%	100.00%
StampMLP-6	99.43%	99.80%	100.00%

**Table 5:** Accuracy of seal surface classification with fewer training images.

	Top-1	Top-3	BG-Top-1
VGG16	80.09%	88.11%	79.50%
ResNet50	52.46%	71.32%	100.00%
MLP-Mixer-3	88.28%	95.59%	100.00%
StampMLP-3	89.61%	96.06%	100.00%

**Table 6:** Accuracy of individual seal classification.

	Top-1*	Top-1	Top-3	BG-Top-1
VGG16	98.70%	90.74%	97.74%	87.50%
ResNet50	93.60%	78.10%	95.03%	48.00%
MLP-Mixer-3	97.57%	85.50%	96.23%	65.00%
MLP-Mixer-6	96.93%	84.25%	95.81%	78.50%
StampMLP-3	98.90%	91.96%	97.87%	98.50%
StampMLP-6	98.41%	91.03%	98.29%	93.00%

**Table 7:** Computational complexity comparison.

	Params	FLOPs	Throughput	MACs	Training Epochs
VGG16	134.60 M	80.51 G	61	2197.84 MB	100
ResNet50	23.67 M	21.47 G	1	1590.33 MB	100
MLP-Mixer-3	3.57 M	1.21 G	986	74.62 MB	48
MLP-Mixer-6	6.91 M	2.21 G	431	141.4 MB	48
StampMLP-3	2.67 M	784.69 M	1132	55.96 MB	48
StampMLP-6	5.33 M	1.57 G	563	109.5 MB	48

**Table 8:** Detailed specifications of the implemented environment.

	Operating System	Programming Language	Server	GPU
specification	CentOS	Python	RTX3090	True

**Table 9:** Accuracy of different product types’ classification with different methods.

Models	VGG16	VGG16-1	VGG16-2	Stamp-MLP-3	Stamp-MLP-6
Accuracy	89.84%	92.71%	99.41%	98.86%	98.31%

**Table 10:** Accuracy of different product types’ classification with different methods.

Models	VGG16	VGG16-1	VGG16-2	Stamp-MLP-3	Stamp-MLP-6
Accuracy	88.67%	91.21%	97.47%	97.87%	98.29%

**Table 11:** Accuracy of different product types’ classification with different methods.

Models	VGG16	VGG16-1	VGG16-2	Stamp-MLP-3	Stamp-MLP-6
Accuracy	94.84%	95.21%	100.00%	99.47%	99.43%

## Data Availability

Not applicable.
